# Perfusion imaging with arterial spin labeling (ASL)–MRI predicts malignant progression in low‑grade (WHO grade II) gliomas

**DOI:** 10.1007/s00234-021-02737-4

**Published:** 2021-06-11

**Authors:** Christina M. Flies, Tom J. Snijders, Tom Van Seeters, Marion Smits, Filip Y. F. De Vos, Jeroen Hendrikse, Jan Willem Dankbaar

**Affiliations:** 1 Department of Neurology & Neurosurgery, UMC Utrecht Brain Center, University Medical Center Utrecht, Heidelberglaan 100, 3584 CX Utrecht, the Netherlands; 2 Department of Radiology, University Medical Center Utrecht, Utrecht, the Netherlands; 3 Department of Radiology, Elisabeth-TweeSteden Ziekenhuis, Tilburg, the Netherlands; 4 Department of Radiology and Nuclear Medicine, Erasmus MC, University Medical Centre Rotterdam, Rotterdam, the Netherlands; 5 Department of Medical Oncology, University Medical Center Utrecht, Utrecht, the Netherlands

**Keywords:** Arterial spin labeling, Low-grade glioma, Malignant progression, Perfusion MRI

## Abstract

**Purpose:**

Predicting malignant progression of grade II gliomas would allow for earlier initiation of treatment. The hypothesis for this single-centre, case–control study was that the perfusion signal on ASL-MRI predicts such malignant progression in the following 12 months.

**Methods:**

Consecutive patients with the following criteria were included: ≥ 18 years, grade II glioma (biopsied or resected) and an ASL-MRI 6–12 months prior to malignant progression (cases) or stable disease (controls). Malignant progression was defined either radiologically (new T1w-contrast enhancement) or histologically (neurosurgical tissue sampling). Three controls were matched with each case. Some patients served as their own control by using earlier imaging. The ASL-MRIs were reviewed by two neuroradiologists and classified as positive (hyper-intense or iso-intense compared to cortical grey matter) or negative (hypo-intense). In patients with epilepsy, a neurologist reviewed clinicoradiological data to exclude peri-ictal pseudoprogression. The statistical analysis included diagnostic test properties, a Cohen’s Kappa interrater reliability coefficient and stratification for previous radiotherapy.

**Results:**

Eleven cases (median age = 48, IQR = 43–50 years) and 33 controls (43, 27–50 years) were included. Malignant progression appeared at 37 months (median, IQR = 17–44) after first surgery. Thirty ASL-MRIs were assessed as negative and 14 as positive. None of the MRIs showed signs of peri-ictal pseudoprogression. ASL significantly predicted subsequent malignant progression (sensitivity = 73%; specificity = 82%; OR = 12; 95%-CI = 2.4–59.1; p = 0.002). The interrater reliability coefficient was 0.65. In stratified analysis, ASL-MRI predicted malignant progression both in patients with previous radiotherapy and in those without (Mantel–Haenszel test, p = 0.003).

**Conclusion:**

Perfusion imaging with ASL-MRI can predict malignant progression within 12 months in patients with grade II glioma.

## Introduction

Diffuse gliomas are the most common adult-onset primary malignant brain tumours [1] and are classified in the World Health Organisation (WHO) 2016 criteria according to histological and molecular-genetic characteristics as grade II, III or IV [2]. Low-grade gliomas (LGGs, WHO grade II) include isocitrate dehydrogenase (IDH)–mutant astrocytomas, IDH-mutant and 1p/19q-codeleted oligodendrogliomas and the more aggressive IDH-wildtype tumours. Overall survival in IDH-mutant LGGs varies from 5 to more than 13 years, depending on patient characteristics, histomolecular subtypes and treatment forms [3, 4].

The duration of survival is largely determined by the occurrence of dedifferentiation, or ‘malignant progression’ (MP). MP of grade II into grade III or IV gliomas inevitably follows after a variable period of time [4]. Predicting MP would permit for earlier initiation of therapy. MP was described in 44 of 216 patients with LGG after a median time of 10.1 years (range for the patients treated with radiotherapy = 19–79 months) and in another observational study, in 124 of 599 patients with LGG after a median time of 4.7 years (range = 1.6–299.7 months) [5, 6]. Given this variable time to MP, predicting the moment of MP in individual patients is difficult.

To identify MP as soon as possible, the surveillance of patients with stable LGG consists of regular MRI, usually with 6-month intervals, based on international guidelines [7]. New or increasing regions of enhancement, defined by the RANO criteria as an increase of the perpendicular diameter of the lesion compared to the post-operative MRI [8], are the most commonly used features to identify MP. MR perfusion techniques may have the potential for early identification of MP. DSC, which relies on an intravascular contrast agent, is the most widely used method [9]. The primary parameter that is determined with DSC perfusion is relative CBV (rCBV). Using DSC, previous authors have reported an increase in rCBV in the 12 months before dedifferentiation compared to patients with stable disease [10]. Another MR perfusion method is arterial spin labeling (ASL). This method does not depend on a contrast agent, but on labelled images with magnetised arterial blood protons [11, 12]. These images are subtracted from control images, containing the same static tissue signals, and CBF maps are generated by averaging many control-label pairs. High relative CBF measurements, as measured with ASL, predicted tumour progression in a cohort of paediatric diffuse astrocytomas, especially in combination with ^18^F–DOPA-PET and ADC [13]. ASL images can also be assessed visually to identify relative areas of hyperperfusion. This method may be easier to use during daily clinical practice than quantification.

Our hypothesis for this single-centre, retrospective case–control study was that hyperperfusion on ASL-MRI in patients with grade II gliomas predicts malignant progression within 12 months.

## Methods

### Patient selection

The institutional Medical Ethical/Biobank Committee approved the use of patient data in the context of an overlapping not yet published study in progress, for which patients had provided written informed consent (protocol no. 16–342/16–340).

For this single-centre, retrospective case–control study, all patients with a WHO grade II glioma from our local database (which started from January 2000) were reviewed in May 2019. Furthermore, patients with a grade III or IV glioma were reviewed to detect secondary malignant tumours after a first surgery in another hospital, because these patients were not included in our local database of patients with a grade II glioma.

The inclusion criteria were (a) aged 18 years or older at first surgery; (b) histologically confirmed WHO grade II glioma according to WHO 2016 criteria (biopsy or resection) [2]; (c) availability of an ASL-MRI (index MRI) performed as part of routine clinical care, from January 2016 until May 2019, and (d) diagnosis of MP for the cases, or stable disease for the controls. MP was defined as new contrast enhancement (CE) on T1w follow-up MRI and/or histological proof of progression to grade III or IV within the subsequent 12 months after the ASL-index MRI, whereas stable disease was defined as no new CE on T1w follow-up MRI and/or a stable grade II lesion at recurrence-resection within the subsequent 12 months after the ASL-index MRI. The presence of MP was determined blinded to the ASL results by a medical researcher (CMF) and a neuro-oncologist (TJS, 11 years experience) based on the radiological, pathological, and multidisciplinary team reports. Based on histomolecular tumour type (including 1p/19q-status and IDH-status), age (2 categories: 18–59 years and 60 years or over), treatment before the ASL and occurrence of epilepsy, three controls were matched with each case. Cases ideally provided their own control with one ASL-MRI followed by 12 months of stable disease and thereafter another one followed by malignant progression with a period of at least 12 months between the two ASL-MRIs. Previous treatment with radiotherapy (RT) was accepted despite the risk of mistaking radiation-induced pseudoprogression for MP. Consequently, only patients with clinicoradiological follow-up of at least 6 months showing further progression and a conclusion of true progression in the multidisciplinary tumour board were included.

### MRI protocol

Pseudocontinuous ASL perfusion MRI is performed in this hospital for the surveillance of LGG on a 3.0 T MRI scanner (Ingenia, Ingenia CX, Achieva, Philips Healthcare, Best, the Netherlands) with the following sequence parameters: repetition time/echo time, 4000/16 ms; flip angle, 90°; field of view, 240 × 240 × 119 mm (RL, AP, FH); section thickness, 7 mm; in-plane voxel size, 3 × 3 × 7 mm; number of slices in the acquisition, 17; WFS (pix)/BW (Hz), 8.254/52.6; post labeling delays, 1525 ms; readout type, 2D EPI; background suppression pulses = on; label duration, 1650 ms; no M0-scan performed; and total scan time, 3 min 19.7 s. The parameters for the three-dimensional T1-weighted imaging include repetition time/echo time, 5.3/2.4 ms; flip angle, 10°; field of view, 230 × 230 × 160 mm (RL, AP, FH); and gap, − 0.5 mm. Patients either underwent 3.0 or 1.5 T MRI for their follow-up, based on availability, so the collection of 3.0 T follow-up MRIs (including ASL) represented a randomly selected subset.

### Analysis

The assessment of all randomly ordered ASL-index MRIs was performed independently by two neuroradiologists (JWD: 10 years experience, TvS: 8 years experience), who were blinded to the outcome and the clinical data. The ASL images were visually graded in three groups: 0 as hypo-intensity, 1 iso-intensity and 2 hyper-intensity, all compared to cortical grey matter on the perfusion-weighted maps. In general, the cortical grey matter in the same hemisphere closest to the glioma was used for this comparison. No other (quantitative) measurements were performed. A crosshair was used on FLAIR images to identify the location of tumour on the ASL-MRI after automatic registration in our PACS.

To reflect clinical practice, one reader’s assessment (by JWD) was used for our main analysis of diagnostic value. The ASL results were dichotomised as positive (grade 1 and 2) or negative (grade 0). As a sensitivity analysis, we repeated calculations with grade 2 scans classified as positive, and grade 0 and 1 scans as negative. The second reading (by TvS) was only used for the interobserver reliability analysis. For this secondary analysis, each ASL-MRI was reviewed by both observers. Given the goal of the second reading, there was no need for a consensus reading, and no consensus reading was performed.

To explore the clinical significance of ASL findings, we registered the interval between the index ASL scan and the next scan. An ASL scan was considered clinically significant when a patient deteriorated clinically before the planned follow-up MRI due to MP. Presumably, the clinical significance of a positive ASL is highest in cases of earlier-than-planned follow-up, especially with clinical deterioration, since early ASL-based identification of MP might prevent such deteriorations.

To exclude peri-ictal pseudoprogression as the cause of radiological changes, a neuro-oncologist (TJS) reviewed clinicoradiological data of patients with seizure symptoms within 3 months previous to a positive ASL blinded to the outcome and excluded MRIs showing signs such as cortical or subcortical enhancement, cortical swelling and diffusion restriction [14] from further analysis. Furthermore, a medical researcher (CMF) reviewed electronic patient files for radiological and histomolecular tumour characteristics, date of first surgery, date of progression and seizure symptoms blinded to the ASL results.

### Statistics

We performed the calculation of an odds ratio (OR) with 95%-confidence interval (95%-CI), sensitivity and specificity for identifying MP. Continuous variables were expressed as median or mean with an interquartile range (IQR) or standard deviation. P-values < 0.05, calculated with the Fisher exact test, were considered statistically significant. To test the robustness, we used the assessment of the second less experienced reader (TvS) to perform an interrater reliability analysis (Cohen’s Kappa coefficient [15]) with the same dichotomised categories as mentioned above (positive versus negative). Since we only included participants with complete information, we did not have missing data. To estimate whether there was an association between ASL and MP independent of whether the patient received previous RT and independent of their IDH-mutational status, the Mantel–Haenszel test was used. No subgroup analysis of cases with histological follow-up (n = 4) versus radiological follow-up was performed due to the small sample size. SPSS (Version 25.0, 2017, IBM SPSS Statistics, Armonk, NY:IBM Corp.) was used for the calculations.

## Results

### Patient characteristics

**Fig. 1:**
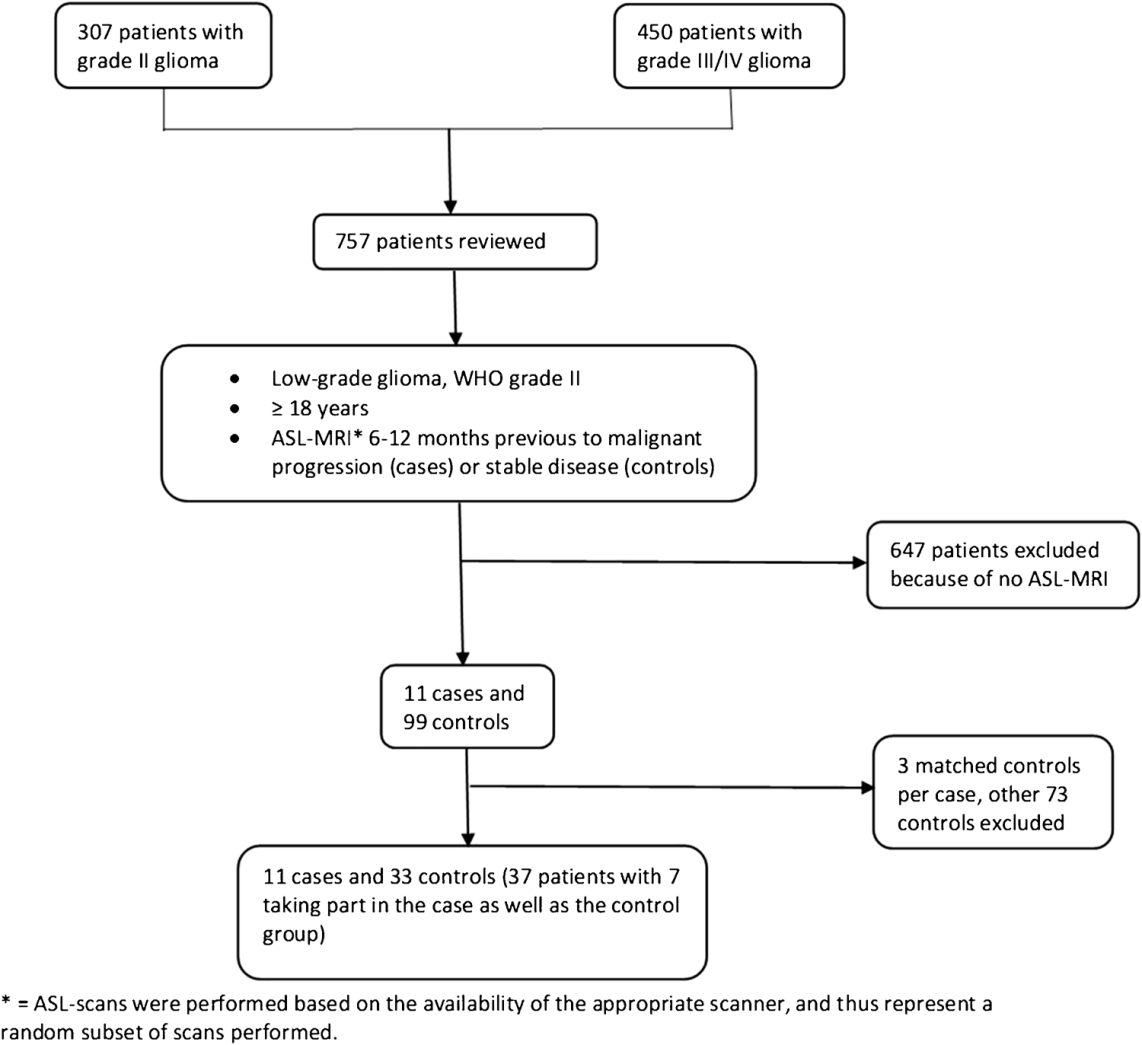
Flowchart of the patient selection process

In total, we identified and reviewed the records of 757 consecutive patients, 307 with a WHO grade II glioma and 450 with a grade III or IV, from which 11 cases and 99 controls were identified. After matching, 11 cases and 33 controls were included in this study; for seven cases, an appropriate control scan was available within the same case. Figure 1 shows the patient selection process. Baseline criteria are given in Table 1. Individual data can be seen in Tables 4 (cases) and 5 (controls) including tumour type, treatment before the ASL, seizures before the ASL and ASL score. For the cases, the time from ASL to MP, from first surgery to MP and from MRI showing MP to second surgery are additionally available in the Appendix. Of these 37 participants, 24 were men and the median age was 45 (IQR 28–50) years with 48 (43–50) years for the cases and 43 (27–50) years for the controls. The majority represented a diffuse astrocytic tumour type. MP occurred at a median of 37 (17–44) months after first surgery. The median interval between the ASL-MRI and the diagnosis of MP was 100 (31–135) days. Treatment before the ASL-MRI included RT and/or temozolomide or procarbazine, lomustine and vincristine chemotherapy. Four cases underwent recurrence resection to confirm the diagnosis, including one without CE on T1w-MRI. In the other seven, progression was confirmed radiologically by new T1w-CE. Controls showed persisting stable disease in the next 12 months. Characteristics of the control sample were matched well, though not perfectly, with their matched cases, reflecting the limitations in the availability of controls. Five patients showed an IDH-wildtype glioma (two cases and three matched controls). This diagnosis was based on IDH-sequencing.

**Table 1:** Baseline criteria

	Cases (11)		Controls (33)	
	Number	Percentage	Number	Percentage
Sex				
Women	5	46	11	33
Men	6	54	22	67
Age in years				
Median, range, IQR	48, 25–57, 43–50		43, 18–57, 27–50	
Histological and molecular tumour type				
Diffuse astrocytoma IDH mt, 1p/19q intact	6	55	23	70
Oligodendroglioma IDH mt, 1p/19q codeleted	1	9	3	9
Glioma IDH wt	2	18	3	9
Glioma not otherwise specified	2	18	4	12
Treatment before ASL-MRI				
No treatment	2	18	11	33
Temozolomide	2	18	1	3
Radiotherapy	2	18	7	21
Radiotherapy and temozolomide	5	46	13	39
Radiotherapy and PCV	0	0	1	3
Time from ASL-MRI to MP in days				
Median, range, IQR	100, 17–203, 31–135			
Time from first surgery to MP in months				
Median, range, IQR	37, 3–116, 17–44			
Seizures preceding ASL: Yes	4		6	
Time between last seizure and ASL in days				
Median, range, IQR	21, 1–75, 4–64		26, 14–79, 19–63	

*IQR*, interquartile range; *IDH wt*, IDH wildtype; *IDH mt*, IDH-mutated; *PCV*, procarbazine/lomustine/vincristine; *MP*, malignant progression; *ASL*, arterial spin labeling MRIs

### Analysis

We encountered no missing data. Of all 44 ASL-MRIs that were analysed, 14 scans were classified as positive, 5 as ‘hyper-intense’ compared to cortical grey matter and 9 as ‘iso-intense’ as seen in Tables 2, 4 and 5. This classification resulted in an OR of 12 (95%-CI 2.4 to 59.1), a sensitivity of 8 of 11, 73%, and a specificity of 27 of 33, 82%, with p = 0.002. The sensitivity analysis with only hyper-intense scans classified as positive, and iso- or hypo-intense scans as negative, resulted in an OR of 2.22 (95%-CI 0.3 to 15.4, p = 0.42). The interrater reliability analysis between the two neuroradiologists produced a kappa value of 0.65, which represented a substantial agreement. Disagreements between raters were seen in every category (true positive, false positive, true negative, false negative).

**Table 2:** Results 1: RT strata

			Malignant progression		P-value	Common OR, 95%-CI, p-value
			No	Yes		
No RT	ASL	Negative	9	0	0.019	
		Positive	3	4		
RT	ASL	Negative	18	3	0.043	
		Positive	3	4		
Total	ASL	Negative	27	3	0.002	15, 2.6–87.7, 0.003
		Positive	6	8		

*RT*, radiotherapy; *ASL*, arterial spin labeling; *OR*, odds ratio

**Fig. 2:**
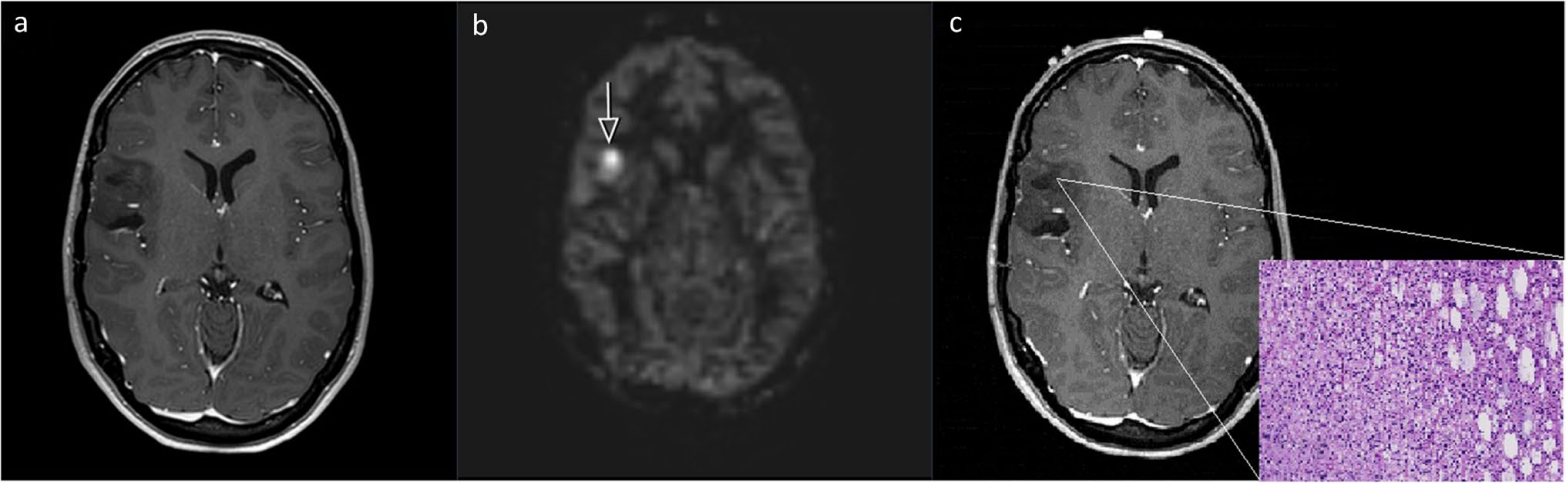
Twenty-three-year-old woman with an untreated diffuse IDH mutated astrocytoma. False-positive scan; **2.a** Index T1-MRI with contrast agent: no contrast enhancement. **2.b** Index ASL rated as positive. **2.c** Follow-up: pre-operative T1-MRI with contrast agent (70 days after index MRI), no contrast enhancement, histology of recurrence resection (H&E staining, × 200) shows a low-grade lesion. All axial planes

**Fig. 3:**
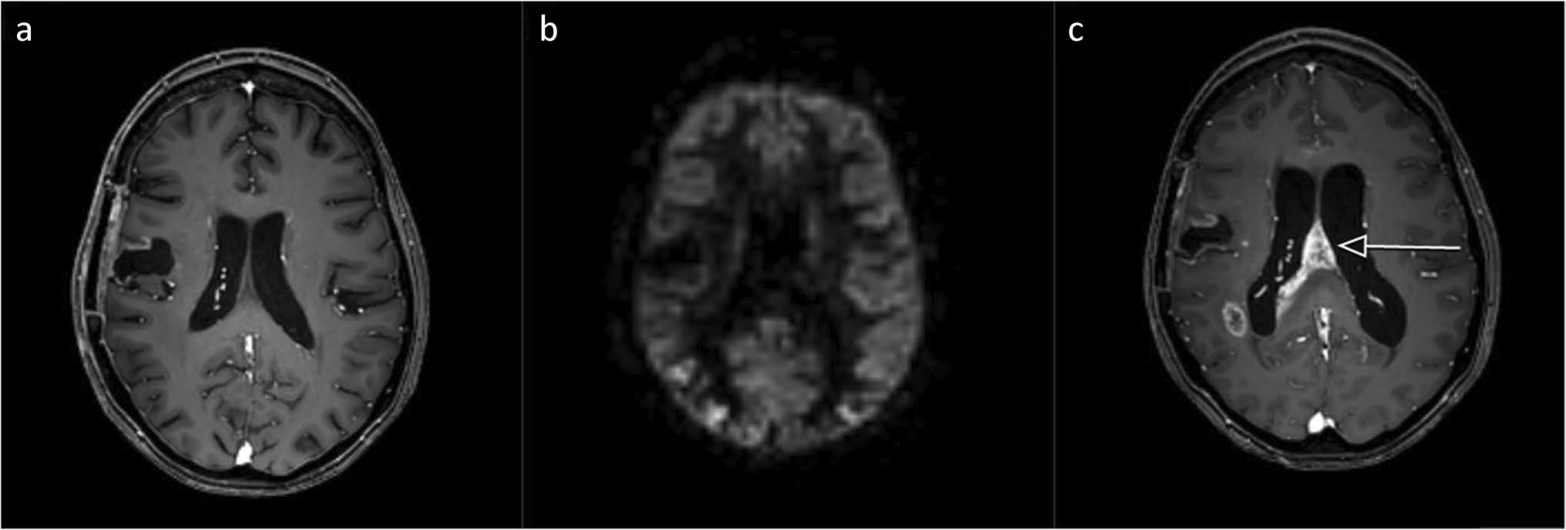
Fifty-five-year-old woman with a diffuse IDH wildtype astrocytoma treated with radiotherapy and temozolomide. False-negative scan; **3.a** Index T1-MRI with contrast agent: no contrast enhancement. **3.b** Index ASL rated as negative. **3.c** Follow-up T1-MRI with contrast agent (203 days after index MRI): new contrast enhancement. All axial planes

**Fig. 4:**
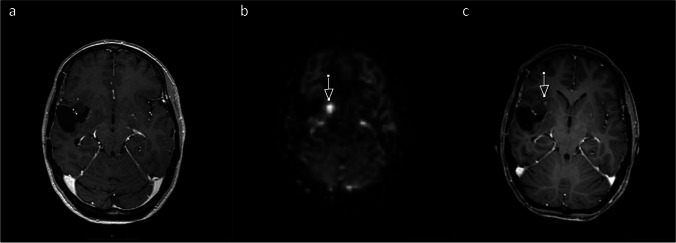
Forty-three-year-old woman with an untreated oligodendroglioma. True positive scan; **4.a** Index T1-MRI with contrast agent: no contrast enhancement. **4.b** Index ASL rated as positive. **4.c** Follow-up T1-MRI with contrast agent (17 days after index MRI): new contrast enhancement. All axial planes

When stratifying the results for previous RT, the pooled estimate of the OR was 15 (95%-CI 2.6 to 87.7, p = 0.003) as seen in Table 2. The stratification for IDH-mutational status produced a common OR of 23.3 (95%-CI 2.4 to 226.8, p = 0.007) as seen in Table 3. Typical examples of ASL scans are presented in Figs. 2, 3 and 4.

**Table 3:** Results 2: IDH strata

			Malignant progression		P-value	Common OR, 95%-CI, p-value
			No	Yes		
IDH wt	ASL	Negative	3	1	0.40	
		Positive	0	1		
IDH mt	ASL	Negative	20	1	0.005	
		Positive	6	6		
Total	ASL	Negative	23	2	0.003	23.3, 2.4–226.8, 0.007
		Positive	6	7		

*IDH wt* = IDH wildtype; *IDH mt* = IDH-mutated; *ASL* = arterial spin labeling; *OR* = odds ratio.

To exclude interfering effects of epilepsy, six patients, three cases and three controls were reviewed, and none of whom showed signs of peri-ictal pseudoprogression.

### Clinical significance of ASL

Of the eight cases with a positive ASL and eventual MP, five underwent follow-up scanning at an earlier time point than planned. In four cases (cases 1, 2, 6 and 7), the T2w abnormalities had already been increasing before the evidence of MP, suggesting low-grade progression, and the decision to treat and to shorten the scan interval had already been taken at the time of the index scan. In the fifth case (case 9), an early follow-up scan was performed solely because of clinical deterioration. Of the total number of 14 patients with a positive ASL-MRI (8 cases and 6 controls), only the 1 case described above met the requirements of a clinically significant scan and none of the six controls, which represented 13% (1 of 8) of the positively rated cases. This rate of earlier-than-planned scans was higher in the true positive group (13%) than the 4% (1 of 27) in the true-negative group.

## Discussion

The aim of this study was to investigate the ability of ASL to detect malignant progression (MP) in patients with low-grade glioma (LGG) before MP becomes visible on T1w-MRI. Predicting MP could be of great clinical value, because appropriate treatment adaptations could be started earlier and therefore more effectively. In this way, clinical deterioration might be prevented, resulting in a longer survival time and better quality of life. In this study, a statistically significant association between ASL positivity and MP within 12 months in LGG was noted (p = 0.002) with a substantial interrater agreement (Cohen’s kappa 0.65). This association persisted after stratification for previous treatment with radiotherapy (OR = 15, p = 0.003) and for IDH-mutational status (OR = 23.3, p = 0.007). Consequently, patients with LGG and a positive ASL should be considered at high risk of impending MP. In these patients, closer follow-up with short imaging intervals could be considered. Currently, in daily clinical practice, ASL positivity alone is not considered sufficient proof of MP.

Previous literature investigating prediction of MP with perfusion MRI is scarce. Danchaivijitr et al. compared the mean value of rCBV at 18, 12 and 6 months before MP between groups with and without MP and reported an increase in the 12 months before [10]. Morana et al. determined a maximum rCBF with ASL-MRI in combination with ^18^F–DOPA-PET and ADC for the detection of progression in 26 children with diffuse astrocytoma [13]. PET-scan and diffusion MRI have previously been used to predict MP in LGG. In one study, the interval change of the 10th percentile ADC predicted progression on average 8 months before the RANO criteria in 12 of 13 patients with progressive LGG compared to 3 of 15 with stable disease [16]. In another study, MP appeared after 2 to 27 months after initial diagnosis in 78% of diffuse LGG with ^18^F-FET-PET uptake shortly after the diagnosis compared to 0% with circumscribed LGG without uptake [17]. More recently, Bashir et al. described 40 ^18^F-FET-PET sessions of 42 patients with LGG showing positive uptake, from which 58% developed MP compared to 1 of 7 in the FET-negative group [18]. ASL-MRI has the advantage over PET and rCBV that ASL can easily be incorporated in standard-of-care follow-up with MRI and does not require the use of a contrast agent.

### Limitations and strengths

Firstly, the small sample size is a result of the recent implementation of ASL-MRIs in daily clinical practice and the low incidence of MP in LGG. Secondly, patients with the rare 1p/19q-codeleted glioma subtype (oligodendroglioma) were scarce. Consequently, the generalisability to the entire LGG population needs to be confirmed in a larger multi-centre consecutive cohort study. Thirdly, previous studies have found higher rCBV in grade II oligodendrogliomas compared to astrocytomas. This may also have influenced our analysis [19, 20]. In our study, the six false-positively rated ASL-MRIs included one of the three oligodendroglioma controls and five of the 30 astrocytoma controls. Fourthly, we included patients with potential radiation necrosis/pseudoprogression, which may be mistaken for MP. However, such misinterpretation should be minimised by the long follow-up period. Fifthly, patients with no ASL-MRI available were excluded, which excluded all patients developing MP before 2016 and with follow-up imaging at another hospital. This could have resulted in a selection bias with more high-risk cases being followed in a university hospital. However, ASL imaging is included routinely in the follow-up of patients with LGG, and therefore, no patients being followed in this hospital after 2016 were excluded. Sixthly, not every progressive glioma shows contrast enhancement, and some patients may not have been recognised having MP in the case of no available histological outcome. However, histological confirmation is not always clinically feasible.

Lastly, ASL-MRI in itself represents a technique with potential for improvement with a relatively low spatial resolution and no defined widely approved quantitative perfusion threshold values. Therefore, we used a visual assessment method in this study, which was judged to be easier to implement in daily clinical practice and without the need for post-processing tools.

Strengths of the study constituted the double, independent radiological assessment blinded to the clinical data, as well as the consideration of peri-ictal pseudoprogression. Furthermore, the prespecified assessment criteria can be named resulting in a substantial interrater agreement. Patients’ tumour types were evaluated according to WHO 2016 criteria, and our results appeared to be independent of IDH-mutational status. To our knowledge, this is the largest study to date to examine the relation between ASL and subsequent MP in LGG.

Future studies are needed to validate our findings, study the specific value of ASL-MRI in the different tumour subtypes and to evaluate the added value of a quantitative analysis.

## Conclusion

Perfusion imaging with ASL-MRI can detect malignant progression before T1-MRI within 12 months in treated and untreated patients with low-grade glioma with a sensitivity of 73% and a specificity of 82%. A positive ASL-MRI during follow-up should therefore be a warning sign and encourage closer follow-up in these patients.

## Data Availability

Individual, anonymised data is included in the Appendix.

## Appendix

Tables 4 and 5

**Table 4:** Baseline characteristics — case patients

Nr	Sex	Age	Tumour type	Treatment preceding ASL	Best response (mo)	Time ASL-MP (d)	Time surgery 1-MP (mo)	Time MRI-surgery 2 (d)	Seizures	ASL
1	W	43	ODG 1p/19q-codel, IDH mt	No	N/A	17	42	No 2^nd^ surgery	Yes	2
2	M	48	DA IDH mt	No	N/A	18	12	No contrast-enhancement on follow-up MRI	No	1
3	M	26	DA IDH mt	TMZ 9 cycles	SD (24)	112	33	No 2^nd^ surgery	No	1
4	W	55	DA IDH wt	RT 50.4 Gy, TMZ 12 cycles	SD (7)	203	17	No 2^nd^ surgery	Yes	0
5	W	45	DA IDH mt	RT 50.4 Gy	SD (36)	100	44	No 2^nd^ surgery	No	0
6	M	57	DA IDH mt	RT 50.4 Gy, TMZ 12 cycles	PR (4)	31	23	No 2^nd^ surgery	No	2
7	W	49	DA IDH mt	RT 50.4 Gy, TMZ 9 cycles	MR (11)	85	116	1	Yes	1
8	M	47	DG-NOS	RT 50.4 Gy	SD (20)	86	37	No 2^nd^ surgery	No	0
9	M	50	DA IDH wt	RT 50.4 Gy, TMZ 14 cycles	PR (17)	135	37	18	No	1
10	W	25	DA IDH mt	RT 50.4 Gy, TMZ 6 cycles	SD (47)	188	111	258	Yes	1
11	M	49	DG-NOS	TMZ 3 cycles	TP	116	3	No 2^nd^ surgery	No	1

Nr, number of case patient; W, woman; M, man; ODG 1p/19q-codel, 1p/19q-codeleted oligodendroglioma; DA, diffuse astrocytoma; IDH, Isocitrate dehydrogenase; mt, mutated; wt, wildtype; DG-NOS, diffuse glioma not otherwise specified; RT, radiotherapy; TMZ, temozolomide; Gy, Gray; Best response: best response to treatment using RANO criteria in months [8]; SD, stable disease; PR, partial response; MR, minor response; TP, tumour progression; N/A, not applicable; Time ASL-MP (d), time from index ASL-MRI to malignant progression in days; ASL, arterial spin labeling MRI; Time surgery 1- MP (mo), time from first surgery to malignant progression in months; Time MRI – surgery 2 (d), time from MRI showing MP to second surgery in days (recurrence-resection), Seizures, seizures before ASL; ASL, score ASL of first radiologist.

**Table 5:** Baseline characteristics — control patients

Nr	Sex	Age	Tumour type	Treatment preceding ASL	Seizures preceding ASL	Score ASL
1	W	43	ODG 1p/19q, codel	No	Yes	1
2	W	49	ODG 1p/19q, codel	No	No	0
3	M	47	ODG 1p/19q, codel	No	No	0
4	M	48	DA IDH mt	No	Yes	0
5	M	50	DA IDH mt	No	Yes	0
6	M	40	DG-NOS	No	No	0
7	M	26	DA IDH mt	TMZ 9 cycles	No	1
8	W	52	DA IDH mt	No	No	0
9	W	38	DA IDH mt	No	No	0
10	M	25	DA IDH mt	RT 50.4 Gy, TMZ 12 cycles	No	0
11	W	44	DA IDH mt	RT 50.4 Gy, TMZ 11 cycles	No	0
12	M	57	DA IDH mt	RT 50.4 Gy, TMZ 12 cycles	No	0
13	W	45	DA IDH mt	RT 50.4 Gy	No	0
14	M	21	DA IDH mt	RT 50.4 Gy	No	0
15	W	21	DA IDH mt	RT 50.4 Gy	No	0
16	M	57	DA IDH mt	RT 50.4 Gy, TMZ 12 cycles	Yes	2
17	M	39	DA IDH mt	RT 50.4 Gy, TMZ 12 cycles	No	0
18	M	18	DA IDH mt	RT 50.4 Gy, TMZ 12 cycles	No	0
19	W	49	DA IDH mt	RT 50.4 Gy, TMZ 9 cycles	No	0
20	M	25	DA IDH mt	RT 50.4 Gy, TMZ 3 cycles	No	0
21	M	33	DA IDH mt	RT 50.4 Gy, TMZ 2 cycles	No	0
22	M	53	DA IDH mt	RT 50.4 Gy	No	1
23	M	32	DG-NOS	RT 50.4 Gy	No	0
24	W	27	DG-NOS	RT 50.4 Gy	No	0
25	M	50	DA IDH wt	RT 50.4 Gy, TMZ 8 cycles	No	0
26	M	31	DA IDH mt	RT 50.4 Gy	No	0
27	W	50	DG-NOS	RT 50.4 Gy, PCV 5 cycles	No	0
28	M	55	DA IDH wt	RT 50.4 Gy, TMZ 7 cycles	No	0
29	M	46	DA IDH mt	RT 50.4 Gy, TMZ 7 cycles	No	0
30	M	26	DA IDH mt	RT 50.4 Gy, TMZ 6 cycles	No	2
31	M	45	DA IDH wt	No	Yes	0
32	M	35	DA IDH mt	No	No	0
33	W	23	DA IDH mt	No	Yes	2

Nr, number of control patient; W, woman; M, man; Age, age at first diagnosis; ODG 1p/19q-codel, 1p/19q-codeleted oligodendroglioma; DA, diffuse astrocytoma; IDH, Isocitrate dehydrogenase; mt, mutated; wt, wildtype; DG-NOS, diffuse glioma not otherwise specified; ASL, arterial spin labeling MRI; Score ASL, of first radiologist; RT, radiotherapy; Gy, Gray; TMZ, temozolomide; PCV, procarbazine/lomustine/vincristine.

## Abbreviations

ASL: Arterial spin labeling
MP: Malignant progression
LGG: Low-grade glioma
RT: Radiotherapy
IDH: Isocitrate dehydrogenase
WHO: World Health Organisation
CE: Contrast enhancement
